# ATP levels influence cell movement during the mound phase in *Dictyostelium discoideum* as revealed by ATP visualization and simulation

**DOI:** 10.1002/2211-5463.13480

**Published:** 2022-09-23

**Authors:** Haruka Hiraoka, Jiewen Wang, Tadashi Nakano, Yasuhiro Hirano, Shinichi Yamazaki, Yasushi Hiraoka, Tokuko Haraguchi

**Affiliations:** ^1^ Graduate School of Frontier Biosciences Osaka University Japan; ^2^ Graduate School of Science Nagoya University Japan; ^3^ Graduate School of Informatics Osaka Metropolitan University Japan

**Keywords:** ATP, cell movement, *Dictyostelium*, differentiation, live‐cell imaging, simulation

## Abstract

Cell migration plays an important role in multicellular organism development. The cellular slime mold *Dictyostelium discoideum* is a useful model organism for the study of cell migration during development. Although cellular ATP levels are known to determine cell fate during development, the underlying mechanism remains unclear. Here, we report that ATP‐rich cells efficiently move to the central tip region of the mound against rotational movement during the mound phase. A simulation analysis based on an agent‐based model reproduces the movement of ATP‐rich cells observed in the experiments. These findings indicate that ATP‐rich cells have the ability to move against the bulk flow of cells, suggesting a mechanism by which high ATP levels determine the cell fate of differentiation.

## Graphical abstract

Cell movement was analyzed during the development of *Dictyostelium discoideum*. During the mound phase, ATP‐rich cells efficiently move toward the center in a spiral manner against the rotational motion of ATP‐poor cells. ATP‐rich cells are located around the center, and eventually differentiate into stalk cells, suggesting that the centripetal movement of ATP‐rich cells determines the cell fate of differentiation.

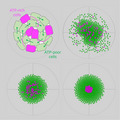

**Abbreviations**
EMCCD: electron multiplying charge coupled device
GFP: green fluorescent protein
RasGAP1: Ras GTPase activating protein 1
RFP: red fluorescent protein

Development is a dynamic process in which cell differentiation occurs with morphogenesis after cells migrate to their destinations [1]. During this process, the regulated migration of cells plays an important role in the normal development of multicellular organisms. The migration of cells toward their destination has been reported in various developmental processes, including gastrulation in early embryogenesis [2, 3], primordial germ cell migration [4], and telencephalon (cerebrum) formation in humans [5]. Errors in the process of cell migration can give rise to serious problems in living organisms. Thus, understanding the regulatory mechanisms of this process is fundamental within the fields of developmental biology and medicine, such as for the study of regeneration therapeutics.

The cellular slime mold *Dictyostelium discoideum* is a good model organism in studies of development and morphogenesis [6, 7]. *D. discoideum* cells usually live as single amoeboid cells in nutrient‐rich conditions (vegetative phase); however, when exposed to starvation, they enter the developmental process and eventually differentiate into only two cell types, namely, stalk cells and spore cells, which go on to form a fruiting body (Figs 1A and S1, Movie S1) [8, 9, 10, 11]. The entire developmental process is completed within approximately 24 h and can therefore be observed under a microscope. Therefore, the movement of cells during development and the accompanying morphological changes are well‐studied [12, 13, 14, 15]. In the early stages of development, approximately 100,000 amoeboid cells move toward the center of the cAMP wave to form an aggregate (aggregation phase) and subsequently a multicellular mound body (mound phase). Cells in the mound body move rotationally around the center of the mound for approximately 4–6 h and differentiate into progenitor cells (called prestalk or prespore cells) during this movement, although they do not show any morphological changes accompanied by differentiation. During this rotational movement, the prestalk cells migrate to the upper center of the mound and eventually become the anterior part of the migrating slug body (slug phase). The slug body moves around until it finds a good environment, after which the prestalk cells and prespore cells in the slug body differentiate into stalk and spore cells to form a fruiting body (fruiting body phase).

**Fig. 1:**
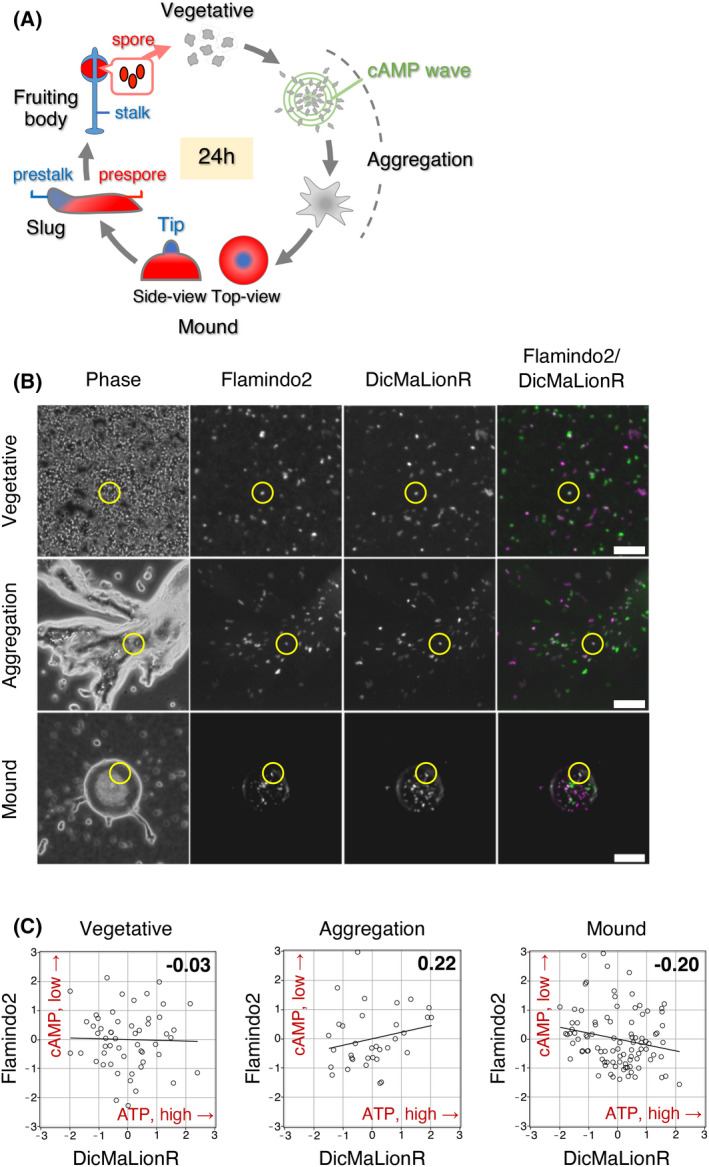
Correlation between ATP and cAMP levels during development (A) Schematic illustration of the developmental process in *Dictyostelium discoideum*. Vegetative cells move toward the center of the cAMP wave to form an aggregate (aggregation phase), subsequently forming a multicellular mound body (mound phase). The cells in a mound body move rotationally around the center, during which they differentiate into two different types of progenitor cells (e.g., prestalk and prespore cells). A slug consists of prestalk cells in the anterior region (blue) and prespore cells in the posterior region (red). Prestalk and prespore cells differentiate into the stalk and spore cells of a fruiting body, respectively. (B) A typical microscopic image of living DicMaLionR/flamindo2 cells during the various developmental phases: vegetative (top), aggregation (middle), and mound (bottom) phases. Phase‐contrast images (leftmost), fluorescence images of flamindo2 (second left), fluorescence images of DicMaLionR (third left), and merged images of flamindo2 and DicMaLionR (rightmost). Time‐lapse images were acquired every 20 s, 20 s, and 1 min in the vegetative, aggregation, and mound phases, respectively. The exposure times for flamindo2 were 200, 200, and 400 ms for the vegetative, aggregation, and mound phases, respectively, while those for DicMaLionR were 100, 100, and 200 ms for the vegetative, aggregative, and mound phases, respectively. Scale bars, 100 μm. (C) Correlation analysis between fluorescence signals of flamindo2 and DicMaLionR at the various developmental phases; vegetative (left), aggregation (middle), and mound (right) phases. The plotted values represent the relative fluorescence intensities of flamindo2 (reciprocal cAMP levels, *y*‐axis) and DicMaLionR (ATP levels, *x*‐axis). The correlation coefficient values are indicated on the upper‐right side of the graph. Number of measurements (*N*) = 54 (vegetative), 34 (aggregation), and 96 (mound).

Various regulatory factors have been reported in the development of *D. discoideum*. cAMP is one such factor, acting as a chemoattractant for cell migration during the aggregation process [16, 17, 18, 19]. The oscillatory wave of cAMP determines the direction of cell migration during the aggregation phase. cAMP is also involved in the coordination of cell sorting [20, 21], pattern formation, and morphogenetic changes [14, 22, 23, 24]. Other factors have been suggested to determine the cell fate of differentiation during development, including the intracellular calcium levels [25, 26, 27, 28], cell cycle [29, 30, 31, 32, 33], and metabolic status [32, 34, 35, 36, 37, 38, 39].

Recently, we have reported that cellular ATP levels during the vegetative phase are responsible for determining cell fate during development [40]: three‐dimensional imaging of live cells revealed that cells with high ATP levels during the vegetative phase tended to congregate at the tip of the mound during the mound phase; ATP‐rich cells at the mound tip remained at the tip (anterior prestalk region) of the slug during the slug phase, and eventually differentiated into stalk cells in the fruiting bodies [40]. Although cAMP is synthesized from ATP in *D. discoideum* [41, 42, 43], the relationship between high ATP levels and cAMP oscillations remains unclear. Furthermore, the mechanism by which ATP determines cell fate during development also remains unclear. In this study, we examined the movement of ATP‐rich cells expressing DicMaLionR as a fluorescent ATP probe in *D. discoideum* and tracked the movement of ATP‐rich cells from the aggregation to the mound phases in the living state. In addition, simulation analysis was performed to reproduce biological experiments.

## Materials and methods

### Plasmid constructs

To construct the plasmid (pDM326‐DicMaLionR and pDM358‐DicMaLionR) encoding an ATP sensor probe for *Dictyostelium*, a DNA fragment encoding codon‐optimized MaLionR, an intensiometric ATP probe [44], was synthesized using GenScript (https://www.funakoshi.co.jp/contents/687) based on the codon usage information for *D. discoideum* (http://dictybase.org). The synthesized DNA fragment was digested with BglII and SpeI and inserted into the blasticidin‐resistant pDM326 vector and hygromycin‐resistant pDM358 vector [45], expressing a coding protein under the *act15* promoter.

### *D. discoideum* strains and cell culture

*Dictyostelium discoideum* strain Ax2 was used as the wild‐type strain for generating DicMaLionR, DicMaLionR/flamindo2, and DicMaLionR/histone H2B‐GFP cell lines. DicMaLionR cells constitutively expressing DicMaLionR were generated as follows: Ax2 cells (1 × 10^7^ cells) were transfected with 1 μg of pDM326‐DicMaLionR plasmid by electroporation using an ECM830 electroporator (BTX, Holliston, MA, USA) (electric conditions: 500 V, 100 μs × 10 pulses, 1‐s interval), and blasticidin‐resistant cells were selected with 10 μg·mL^−1^ of blasticidin. DicMaLionR/flamindo2 cells constitutively co‐expressing DicMaLionR and flamindo2 under *act15* promoter were generated as follows: flamindo2 cells were transfected with 1 μg of pDM326‐DicMaLionR plasmid as described above, and drug‐resistant cells were selected with 10 μg·mL^−1^ of blasticidin and 20 μg·mL^−1^ of neomycin. Flamindo2 cell line was a generous gift from Dr. Ueda (Osaka University) [46, 47, 48]. DicMaLionR/histone H2B‐GFP cells constitutively co‐expressing DicMaLionR and histone H2B‐GFP were generated as follows: histone H2B‐GFP cells were transfected with 1 μg of pDM358‐DicMaLionR plasmid as described above, and drug resistance cells were selected with 10 μg·mL^−1^ of blasticidin and 20 μg·mL^−1^ of hygromycin. Cells from a single clone were used in the experiments.

The cells were maintained at 22 °C in HL5 medium supplemented with 10 μg·mL^−1^ of streptomycin/penicillin solution (168‐23191; FUJIFILM Wako, Osaka, Japan) to prevent contamination.

### Induction of cell development

Cells (Ax2 wild‐type or DicMaLionR/flamindo2 cells or DicMaLionR/histone H2B‐GFP cells) were washed twice with potassium phosphate buffer (KK2: 16.1 mM of KH_2_PO_4_, 4.0 mM of K_2_HPO_4_, pH 6.1) and suspended at a density of 1 × 10^6^ cells·mL^−1^ in KK2 buffer. The DicMaLionR/flamindo2 cells were then mixed with wild‐type cells at a final concentration of 5% of total cells. Then, 1 mL of the cell suspension (1 × 10^6^ cells) was spread on an agar plate to analyze the process of development. One milliliter of 1% Bacto Agar (214010; BD Biosciences, Franklin Lakes, NJ, USA) dissolved in distilled water was plated on a 35‐mm plastic dish as a platform for cell differentiation.

### Live‐cell fluorescence imaging of the developmental process

Live‐cell fluorescence imaging was performed using an IX83 microscope (Olympus, Tokyo, Japan). Images were acquired with a DP80 camera (Olympus, Tokyo, Japan) using a 10× (UPlanFL N 10×/0.3 NA, Olympus, Tokyo, Japan) objective lens through the filter set U‐FGW for DicMaLionR and U‐FBN A for flamindo2. CellSens equipped with a microscope was used to obtain and process the images.

### Cell tracking

Cell tracking analysis was performed using MTrackJ version 1.5.1 software with the default settings [49]. The tracking time period was 30 min with a tracking interval of 1 min.

### Treatment with inhibitors of ATP production

Vegetative DicMaLionR cells were seeded on a 35‐mm glass‐bottom dish (P35G‐1.5‐14‐C, MatTek, Ashland, MA, USA) and washed twice with KK2 buffer after attachment, then filled with KK2 buffer for observation. Five‐minutes later from the start of observation, oligomycin (mixture of A, B, and C isomers; O‐500, Alomone Labs, Jerusalem, Israel), an inhibitor of ATP production, was added at the final concentration of 2.5 μM. Time‐lapse images were acquired every 10 s using 60× oil immersion (PlanApo 60×/1.40 NA, Nikon, Tokyo, Japan) objective lens and Dragonfly200 (Andor, Tokyo, Japan) equipped with EMCCD (iXonUltra 888, Andor, Tokyo, Japan) camera. A solid‐state laser (ILE‐400, Andor, Tokyo, Japan) was used as the source for providing 561‐nm (RFP excitation) wavelength lights.

### Quantification and statistical analysis

Statistical analyses were conducted using the MAPLE 2019 software (Waterloo Maple Inc., Ontario, Canada). Pearson's correlation coefficient was used to examine correlations. Their significance was tested based on the Pearson's correlation table. Fluorescence intensities are scaled under the assumption that they are distributed according to the standard normal distribution. Using the mean, μ, and variance, $\sigma^{2}$, of fluorescence intensities, each intensity, X, is scaled to $\frac{X-\mu }{\sigma }$. A linear regression model using the least‐squares method was used to fit the data.

### Computer simulation model

An agent‐based model was used to reproduce the collective rotational cell motion observed in the mound phase of *Dictyostelium* in microscopic experiments. In the model, a set, N, of cells formed a mound in a two‐dimensional space, where cell, i∈N, at time, t, was characterized by its location vector, $x_{i}\left(t\right)$, and velocity vector, $v_{i}\left(t\right)=\frac{dx_{i}\left(t\right)}{dt}$. Based on Newton's second law of motion, we derived equations of motion for these cells. We then considered the overdamped limit of the equations and neglected the inertial effects, which resulted in the following calculation:

$$\alpha_{i}\;v_{i}\left(t\right)=F_{i}^{\text{cent}}\left(t\right)+F_{i}^{\text{cont}}\left(t\right)+F_{i}^{\mathrm{rep}}\left(t\right).$$

On the left‐hand side of the above equation, $\alpha_{i}\;v_{i}\left(t\right)$ represents the resistance to the cell motion, where $\alpha_{i}$ is a coefficient. On the right‐hand side of this equation, $F_{i}^{\text{cent}}\left(t\right),F_{i}^{\text{cont}}\left(t\right),\text{and}\;F_{i}^{\mathrm{rep}}\left(t\right)$ represent three types of forces that cell i used to produce motion at time t, namely, central, contact‐following, and repulsion forces. A similar force‐based model is used to model collective cell behavior in biology [50]. In the following section, we describe the details of the model used in the simulations.

The resistance force, $\alpha_{i}\;v_{i}\left(t\right)$, represents the resistance to cell motion. As the expression shows, the resistance force increased as cell i's velocity, $v_{i}\left(t\right)$, increased. Furthermore, in this model, the resistance force increased as cell i ‘s local cell density (or the number of nearby cells) increased. Let $M_{i}\left(t\right)$ denote the set of cells within distance $l^{\mathrm{loc}}$ from cell i at time t. We then express coefficient $\alpha_{i}$ as

$$\alpha_{i}=\alpha_{0}+\beta \frac{|M_{i}\left(t\right){|}^{q}}{m^{q}+|M_{i}\left(t\right){|}^{q}},$$

where $\alpha_{0},\beta ,m$, and q are parameters. The second term on the right‐hand side of the above equation is in the form of the Hill equation. $\alpha_{i}=\alpha_{0}$ when $|M_{i}\left(t\right)|=0$. It increased and approached $\alpha_{0}+\beta$ as $|M_{i}\left(t\right)|$ increased. m and q determine how $\alpha_{i}$ increases with $|M_{i}\left(t\right)|.$

The central force, $F_{i}^{\text{cent}}\left(t\right)$, represents the force that cell i at time t used to move toward the mound center. For simplicity, in the model, the mound center was fixed at the origin of the two‐dimensional space, and the cAMP signals were assumed to propagate from the mound center. Based on the propagating cAMP signals, each cell moved toward the origin of the space. The magnitude of this force is given as $k^{\text{cent}};$ and thus,

$$F_{i}^{\text{cent}}\left(t\right)=-k^{\text{cent}}\frac{x_{i}\left(t\right)}{¦x_{i}\left(t\right)¦}.$$

Note that when the cell is at the mound center, $F_{i}^{\text{cent}}\left(t\right)=0$. In computer simulations, we assumed that $k^{\text{cent}}$ is dependent on the cell's intracellular ATP level; ATP‐rich cells had a larger value of $k^{\text{cent}}$ than ATP‐poor cells.

The contact‐following force, $F_{i}^{\text{cont}}\left(t\right)$, represents the force that cell i at time t used to follow other cells with which the cell was in contact. This force allows a group of nearby cells to exhibit collective motion [51]. In the model, two cells i and j were considered to be in contact if they were within a distance $l^{\text{cont}}$ from each other, and the set of cells in which cell i was in contact at time t, excluding cell i itself, is denoted as $C_{i}\left(t\right)$. The magnitude of the velocity is given by constant $\left(k^{\text{cont}}\right)$, and the force is given by

$$F_{i}^{\text{cont}}\left(t\right)=k^{\text{cont}}\sum_{j\in C_{i}\left(t\right)}v_{j}\left(t\right)/¦\sum_{j\in C_{i}\left(t\right)}v_{j}\left(t\right)¦.$$

Note that, under the conditions where the denominator of the right‐hand side of the equation becomes zero, $F_{i}^{\text{cont}}\left(t\right)=0$. In computer simulations, we assumed that $k^{\text{cont}}$ is dependent on the cell's intracellular ATP level; ATP‐rich cells had a smaller value of $k^{\text{cont}}$ than ATP‐poor cells.

The repulsion force, $F_{i}^{\mathrm{rep}}\left(t\right)$, represents the force that cell i at time t used to move away from its nearby cells due to volume exclusion effects. As each cell occupies physical space, the two cells cannot be too close to each other. A similar force has been previously considered when modeling the collective cell motion of *Dictyostelium* [52]. For simplicity, we used $M_{i}\left(t\right)$ to express the set of cell i nearby cells, and $k^{\mathrm{rep}}$ to express the maximum magnitude of the repulsion force that cell can receive from another cell. ican receive from another cell. The repulsion force represents the sum of the forces from all nearby cells, and is given as follows:

$$F_{i}^{\mathrm{rep}}\left(t\right)=-k^{\mathrm{rep}}\sum_{j\in M_{i}\left(t\right)}\frac{l^{\mathrm{loc}}-¦x_{j}\left(t\right)-x_{i}\left(t\right)\;¦}{l^{\mathrm{loc}}}\;\frac{x_{j}\left(t\right)-x_{i}\left(t\right)\;}{¦x_{j}\left(t\right)-x_{i}\left(t\right)\;¦}.$$

Here, the denominator of the right‐hand side of the equation is assumed to be nonzero.

### Computer simulations

Discrete‐time computer simulations were conducted using an agent‐based model. The time was discretized by Δt=0.1 min and the model equations were solved using the Euler method. The number of cells was 1000. Parameter values were unknown and arbitrarily chosen to reproduce microscopic observations: $k^{\text{cent}}=3.4$ ng·μm·min^−2^, $k^{\text{cont}}=18$ ng·μm·min^−2^, $k^{\mathrm{rep}}=7.2$ ng·μm·min^−2^, $l^{\mathrm{loc}}=15$ μm, $l^{\text{cont}}=10\;$μm, $\alpha_{0}=1\;\mathrm{ng}\cdot$min^−1^, β=5ng·min^−1^, m=30 and q=6.

In the computer simulations, the cells were initially placed at randomly selected positions within a two‐dimensional circular space and assigned zero initial cell velocity vectors. Each simulation was terminated after 5 h, during which the cell positions and velocities were recorded every 1 min.

## Results

### Cellular ATP levels are not correlated with cAMP levels during development

To measure intracellular ATP levels in *Dictyostelium*, we generated a red‐emitting fluorescent protein DicMaLionR with modification of DicMaLionG, which has been established as an ATP sensor probe in *Dictyostelium* [40]. DicMaLionR and DicMaLionG have the same ATP‐sensing domain. Oligomycin, an inhibitor of ATP synthesis, reduced fluorescence levels in the cells constitutively expressing DicMaLionR (Fig. S2), suggesting that DicMaLionR acts as an ATP sensor. To further confirm the capability of DicMaLionR as an ATP sensor, the developmental process was observed in cells expressing DicMaLionR (Fig. S3). The results showed that the cells displaying high levels of DicMaLionR fluorescence (high ATP levels) tend to congregate at the central tip region of the mound (Fig. S3B), which was similar to results reported for DicMaLionG and DicAT1.03NL [40]; in contrast, the control cells expressing high levels of mRFPmars did not congregate at the central tip region of the mound (Fig. S3A). These results suggest that DicMaLionR acts as an ATP sensor in *Dictyostelium*. We also used flamindo2, a green‐emitting (reciprocal) cAMP probe [46], to monitor the cAMP levels.

Using these probes, we examined the relationship between intracellular ATP and cAMP levels in *Dictyostelium discoideum* cells during development, as cAMP is synthesized from ATP [41, 42, 43]. Cells expressing both DicMaLionR and flamindo2 (DicMaLionR/flamindo2 cells) were used for this purpose. In these cells, the developmental process proceeded with normal timing and morphology. High levels of DicMaLionR fluorescent signal were observed in the center of the mound formed from DicMaLionR/flamindo2 cells (Fig. S4A), and oscillatory waves of flamindo2 (reciprocal of cAMP level) were observed (Fig. S4B; Movie S2), as reported previously [47, 48].

The DicMaLionR/flamindo2 cells were mixed with nonfluorescent wild‐type cells at a 5% ratio of total cells and induced to development. Time‐lapse images were obtained of living cells at various developmental stages using a fluorescence microscope (Fig. 1B). Cells were randomly selected from the time‐lapse images, and the changes in the fluorescence intensity of DicMaLionR and flamindo2 in these cells were measured for 30 min (Fig. S5). The average intensity of DicMaLionR (ATP) for each cell was plotted against the average intensity of flamindo2 (cAMP, reciprocal) to examine their correlation (Fig. 1C). The results showed that no significant correlation was observed between ATP and cAMP levels in the cells at any phase (Fig. 1C).

Next, we examined whether the ATP levels oscillated in response to cAMP oscillation. Although oscillations in the cAMP level were observed at the aggregation and mound phases, no apparent oscillations in the ATP level were observed during the vegetative, aggregation, and mound phases under the same conditions (Fig. S5). This suggests that no ATP oscillations occur during these developmental phases or that the fluctuation in ATP levels is too small to be detected by the ATP probe. The intracellular ATP level of *D. discoideum* is approximately 1000 times higher than that of cAMP (approximately 1–1.5 mM ATP vs. 1–20 μM cAMP) [53, 54]. Therefore, it is not considered that an increase in the cAMP level directly decreases the ATP level. Although the molecular mechanisms that determine the ATP levels remain unclear, our findings suggest that cAMP oscillations are not a direct, immediate determinant of the oscillatory changes in ATP levels.

### Cells with high ATP levels show high motility to the mound center

**Fig. 2:**
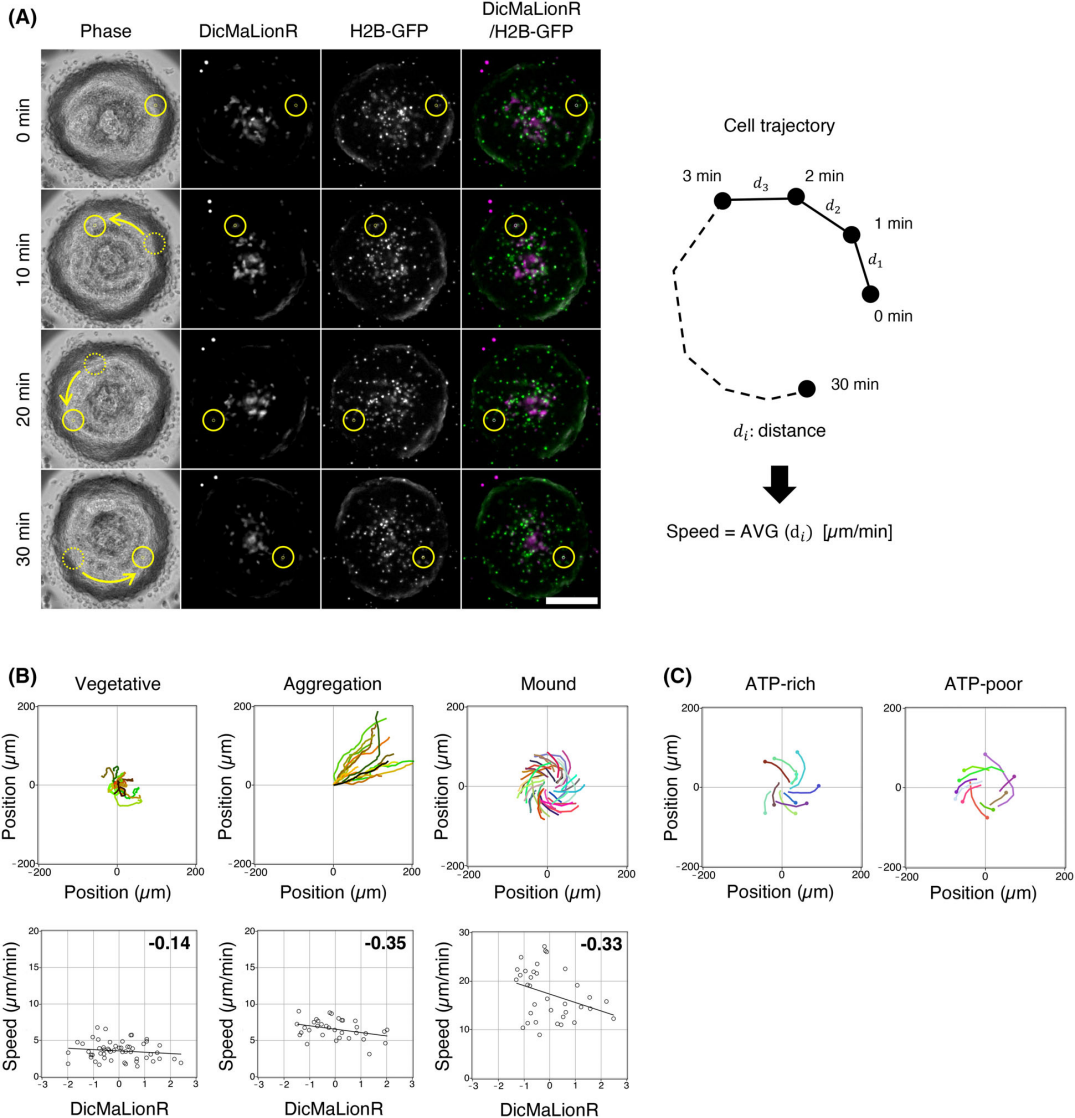
Correlation between the ATP levels and cell migration speed. (A) A typical microscopic image of living DicMaLionR/histone H2B‐GFP cells during the mound phase. Phase‐contrast images (leftmost), fluorescence images of DicMaLionR (second left), fluorescence images of histone H2B‐GFP (third left), and merged images of DicMaLionR and histone H2B‐GFP (rightmost). Yellow‐circled cells show typical examples used for tracking. Dashed yellow circles and arrows represent the past cell position and migration route. Scale bar, 100 μm. Drawings on the right indicate the conceptual illustration of cell trajectory. *D*_*ι*_ represents the migration distance per min in micrometer. Cell speed was calculated based on the equation shown in the illustration. AVG, average. (B) Examples of the tracking of particular cells of interest during developmental phases: vegetative (left), aggregation (middle), and mound (right) phases. The top panels show a tracking map of the positions of the cells. Each color represents the tracking path of a single cell. The bottom panels represent the relationship between the fluorescence intensities of DicMaLionR (ATP levels, *x*‐axis) and cell migration speeds (*y*‐axis) in the cells during each developmental phase. A single spot in the graph represents the value obtained for a single cell. The correlation coefficient values are indicated on the upper‐right side of the graph. *N* = 54 (vegetative), 34 (aggregation), and 35 (mound). (C) Typical tracking paths of ATP‐rich (left) and ATP‐poor (right) cells at the mound phase. Ten cells with higher and lower ATP levels were selected from the trajectory maps in (B).

The correlation between cell motility and ATP levels during the vegetative, aggregation, and mound phases was analyzed. To this end, cells were randomly selected from time‐lapse images of living cells, and their migration speeds were calculated from cell trajectories and plotted against DicMaLionR intensity (ATP level; Fig. 2A). The results showed no significant correlation at the vegetative phase and a weak negative correlation in the aggregation and mound phases (Fig. 2B). This suggests that ATP‐rich cells move more slowly than ATP‐poor cells during the aggregation and mound phases. Moreover, ATP‐rich cells and ATP‐poor cells were found to have different trajectories in the mound phase; ATP‐rich cells had a stronger tendency to move toward the mound center than ATP‐poor cells (Fig. 2C).

**Fig. 3:**
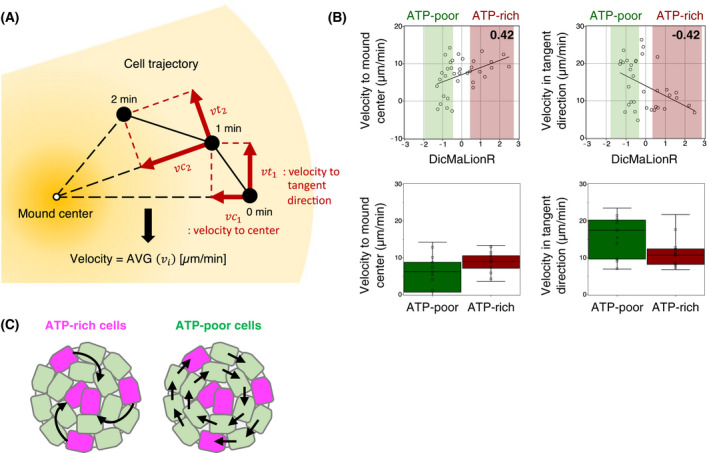
ATP‐rich cells move faster toward the mound center (A) Conceptual cell trajectory. Black dots represent cell positions in cell trajectory. Red arrows indicate the cell velocities to the mound center $\left({\mathrm{vc}}_{i}\right)$ and to the tangential direction $\left({\mathrm{vt}}_{i}\right)$. The equation is shown at the bottom. (B) The top panels show the correlation between the ATP levels and the cell velocity in the center direction (left) and the tangential direction (right). The ATP levels were measured by fluorescence intensity of DicMaLionR. The correlation coefficient values are indicated on the upper‐right side of the graph. *N* = 35. The bottom panels show the comparison results of velocities between ATP‐rich cells (relative ATP levels > 0.5) and ATP‐poor cells (relative ATP levels < −0.5); the cells are shown in the red and green boxes in the top panel, respectively. The results are presented as box‐and‐whisker plots. Boxes indicate the median and the upper and lower quartiles, while the whiskers indicate the range. *N* = 11 (ATP‐rich) and 14 (ATP‐poor). (C) Schematic illustration of the typical movement of ATP‐rich (magenta) and ATP‐poor (green) cells at the mound phase. Black arrows show the typical movement of cells in ATP‐rich (left) and ATP‐poor (right) cells.

Thus, we then examined the difference in the direction of cell migration during the mound phase as this difference may determine the fate of ATP‐rich cells and, therefore, the cell fate of differentiation. For this purpose, we analyzed the velocities of cells in the peripheral area of the mound by separating them into two migration directions, the velocity toward the mound center and the velocity in the tangential direction, and examined the correlation between the velocities and ATP levels (Fig. 3A). The results showed a significant positive correlation between centripetal velocity and the ATP levels (Fig. 3B, upper left), whereas a significant negative correlation was observed for tangential velocity (Fig. 3B, upper right). To directly compare the velocities of ATP‐rich and ATP‐poor cells, ATP‐rich cells were selected as cells with ATP levels higher than 0.5 and ATP‐poor cells were selected as those lower than −0.5, and their velocities were determined. The centripetal velocities were 8.8 ± 3.0 μm·min^−1^ in ATP‐rich cells and 5.4 ± 5.3 μm·min^−1^ in ATP‐poor cells, while their tangential velocities were 11.0 ± 4.2 μm·min^−1^ in ATP‐rich cells and 15.4 ± 5.9 μm·min^−1^ in ATP‐poor cells, indicating that ATP‐rich cells have larger centripetal velocity and smaller tangential velocity than ATP‐poor cells (Fig. 3B, lower panel). This tangential velocity was equivalent to the velocity of collective cell migration with cell–cell contact, as cells in the mound regularly rotate with a constant velocity by interacting with each other. This result indicates that cells with higher ATP levels can move faster to the mound center than those with lower ATP levels (Fig. 3C), suggesting that ATP‐rich cells have a strong driving force to move toward the mound center without participating in collective cell migration. These findings support the model that ATP‐poor cells move rotationally by cell–cell contact and ATP‐rich cells cut off cell–cell contact to move toward the mound center (Fig. 3C). Because cells at the mound center are known to subsequently differentiate into stalk cells [8], this movement toward the mound center is important for cell fate determination.

### Simulation of cell movements produces a pattern similar to the biological experiments

To further prove that accelerated cell motility to the mound center may be a factor responsible for cell fate determination, we conducted computer simulations using a two‐dimensional agent‐based model in which each agent represents a cell and moves based on three types of forces: central force ($F^{\text{cent}}$), contact‐following force ($F^{\text{cont}}$), and repulsion force ($F^{\mathrm{rep}}$). The central force represents the force that a cell uses to move toward the mound center. For simplicity, in the model, the mound center was fixed at the origin of the two‐dimensional simulation space, to which each cell used the central force to move. The contact‐following force represents the force generated by cell–cell contact. This force allows nearby cells to move toward the same direction, leading to the emergence of the collective rotational motion of cells [51]. The repulsion force represents the force that a cell receives from other cells occupying the same physical space, allowing the cell to move away from the other cells.

In computer simulations, we consider cases in which ATP‐rich cells and ATP‐poor cells have different central forces and/or contact‐following forces. For convenience, we define the relative magnitude $w^{\text{cent}}\;$ of the central force. The magnitude of the central force of ATP‐poor cells was $k^{\text{cent}}$, while that of ATP‐rich cells was $k^{\text{cent}}\;w^{\text{cent}}$ $\left(w^{\text{cent}}>1\right)$. We also defined the relative magnitude $w^{\text{cont}}\;$ of the contact‐following force: the magnitude of the contact‐following force of ATP‐poor cells was $k^{\text{cont}}$, while that of ATP‐rich cells was rich cells was $k^{\text{cont}}w^{\text{cont}}$ $\left(w^{\text{cont}}<1\right)$.

**Fig. 4:**
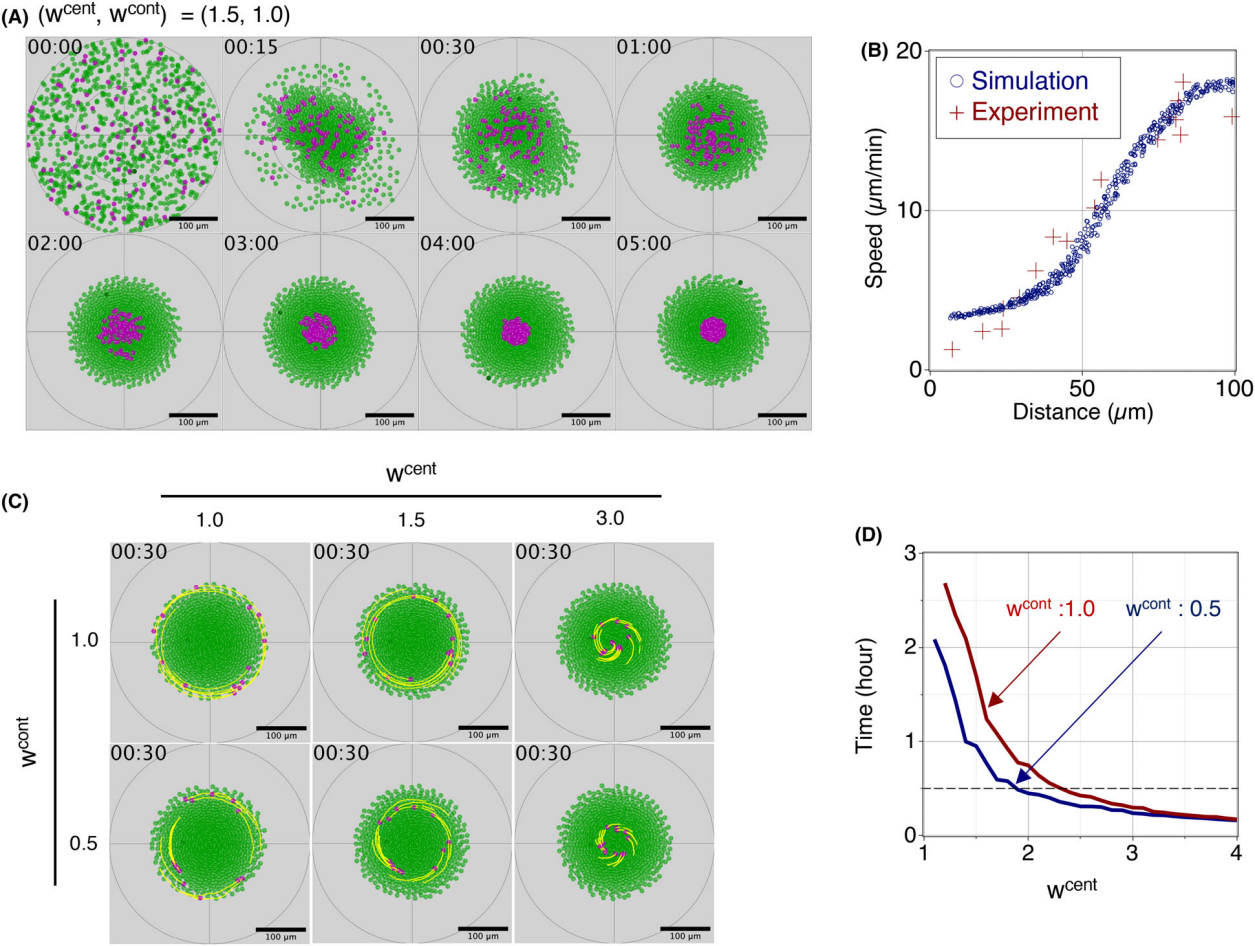
Simulation analysis of collective rotational motion (A) Initially, a total of 1000 cells, including 100 ATP‐rich cells (magenta) and 900 ATP‐poor cells (green), were distributed randomly in the circular region. The relative magnitude of central force ($w^{\text{cent}}$) and contact‐following force ($w^{\text{cont}}$) of ATP‐rich cells against ATP‐poor cells is 1.5 and 1.0, respectively. The number on the upper‐left side of each image indicates the time as “hours:minutes”. ATP‐rich cells then moved toward the center of the region in a spiral manner, while ATP‐poor cells maintained a certain distance from the center of the region and continued rotational motion. In 3 h, cell‐sorting was completed. ATP‐rich cells were located around the center of the region and surrounded by ATP‐poor cells. Scale bars, 100 μm. (B) After cell‐sorting was completed, the moving speed of the cells was found to be an increasing function of a distance from the mound center, which is consistent with the experimental observations. Blue circles and red crosses represent the simulative and experimental data sets, respectively. (C) Impact of the relative magnitudes of central ($w^{\text{cent}}$) and contract‐following forces ($w^{\text{cont}}$) on the locations of ATP‐rich cells (magenta) and their trajectories (yellow). ATP‐poor cells are shown in green. The number on the upper‐left side of each image indicates the time as “hours:minutes”. The time period shown here is 30 min after the mound is formed. Scale bars, 100 μm. (d) Time taken for ATP‐rich cells to move from the edge of the mound to the center of the mound as a function of the relative magnitude of central force ($w^{\text{cent}}$). The relative magnitude of contact‐following force ($w^{\text{cont}}$) is 1.0 (red) and 0.5 (blue). The gray dotted line represents the aggregation time measured from actual data in biological experiments (Fig. 2).

In the computer simulations, we assumed a population of 1000 cells consisting of 100 ATP‐rich cells and 900 ATP‐poor cells. To reproduce the size of the mound observed in biological experiments, the number of cells, the percentage of ATP‐rich cells, and other parameter values in the simulations were determined. Time‐lapse imaging of living cells showed that ATP‐rich cells moved faster to the mound center, suggesting that ATP‐rich cells generated a stronger central force than ATP‐poor cells. Here, we used $w^{\text{cent}}=1.5\;$and $w^{\text{cont}}=1.0$ for the simulation. All 1000 cells had zero initial velocities and moved according to the model over time (Fig. 4A, Movie S3). From 0 to 30 min, the cells moved in an incoherent manner. After 30 min, the cells started to show coordinated rotational motion, moving along the circumference of a circle whose center position coincided with the mound center. While the cells showed this collective rotational motion, ATP‐rich cells were located around the mound center and surrounded by ATP‐poor cells, as shown in Fig. 4A (the cells at “3:00”). Furthermore, in this phase of collective and rotational cell motion, the average moving speed of cells was found to be an increasing function of the distance from the mound center (Fig. 4B), which is in agreement with the data obtained from the biological experiments.

In biological experiments using living cells, we observed that cells moved from the mound periphery to the center in a semi‐spiral manner (Fig. 2A). To examine how ATP‐rich cells in our model moved from the mound periphery to the center, we conducted another set of computer simulations. We ran computer simulations for 2 h to produce collective and rotational cell motion only with ATP‐poor cells and introduced ATP‐rich cells at the mound periphery at 2 h. The results showed that ATP‐rich cells moved toward the mound center in a spiral manner, as observed in the biological experiments. We also examined how the relative magnitude of the central force affects the time taken for ATP‐rich cells to reach the mound center. The mound center was defined as a region within 50 μm of the origin of the two‐dimensional space. The results showed that ATP‐rich cells reached the mound center in a shorter period of time as $w^{\text{cent}}$ increases (Fig. 4C, Movies S4‐S9).

Prespore and prestalk cells may have different contact‐following forces and chemotactic forces [24]. Therefore, we further examined how $w^{\text{cent}}$ affects the time taken to reach the mound center when ATP‐rich cells and ATP‐poor cells have different contact‐following forces ($w^{\text{cont}}=0.5)$. The results showed that ATP‐rich cells reached the mound center in a shorter period of time for any $w^{\text{cent}}$ when $w^{\text{cont}}$ decreased from 1.0 to 0.5 (Fig. 4C). When $w^{\text{cont}}=1.0,$ATP‐rich cells reached the mound center in 102 min for $w^{\text{cent}}=1.5\;$and in 18 min for $w^{\text{cent}}=3.0$ (red line in Fig. 4D, Movies S5 and S6). When $w^{\text{cont}}=0.5,$ on the other hand, the cells reached the mound center in 57 min for $w^{\text{cent}}=1.5$ and in 14 min for $w^{\text{cent}}=3.0\;$(blue line in Fig. 4D, Movies S8 and S9). The difference in the contact‐following force promoted the aggregation of ATP‐rich cells around the mound center. These simulation results demonstrate that the relative magnitudes of the central and contact‐following forces play an important role for determining the centripetal motion of ATP‐rich cells. In biological experiments, ATP‐rich cells reached the mound center within 10–20 min (Fig. 2C), suggesting that the central force of ATP‐rich cells was approximately three times larger than that of ATP‐poor cells under the conditions tested. In addition, the weaker contact‐following force of ATP‐rich cells compared to ATP‐poor cells is indicative of the efficient aggregation of ATP‐rich cells around the mound center.

## Discussion

In this study, the correlation between ATP levels and various factors, such as cAMP, cell motility, and direction of cell migration, were investigated to determine the underlying reasons behind the aggregation of ATP‐rich cells in the central tip region of the mound, before differentiating into stalk cells. We identified that, the migration of ATP‐rich cells toward the center of the mound in opposition to the mass rotational movement of the mound during the mound phase, was one contributing factor to their aggregation (Fig. 3).

Chemotaxis to cAMP might be one possible driving force for the migration of ATP‐rich cells to the mound center, because ATP is the primary source of cAMP generation [41, 42, 43] and cAMP waves from the mound center have been detected during rotational cell movement during the mound phase in *D. discoideum* [48, 52]. However, in this study, simultaneous measurement of intracellular cAMP and ATP levels revealed no apparent correlation between them (Figs 1C and S5). To date, the involvement of cAMP in the centripetal migration of ATP‐rich cells remains unclear. However, it is known that cAMP signaling affects many ATP‐dependent events, such as cell shape alterations [53, 54], actin polymerization [55, 56], intracellular activation of several protein kinases [57, 58, 59], and changes in gene expression [33, 60], suggesting that cAMP may regulate the behavior of ATP‐rich cells through these processes.

Next, we investigated the contribution of cellular ATP to cell motility. ATP is a molecule that provides the energy needed for the biological activities of all living organisms. High levels of ATP may be crucial for particular biological events that require large amounts of energy, such as cell migration [61]. In cancer cells, changes in the intracellular ATP levels have been found to be correlated with changes in cell velocity during migration [62]. In *D. discoideum* , however, no significant correlation between the ATP levels and cell migration speed was observed in the cells during the vegetative phase (Fig. 2B). Rather, the ATP levels and cell migration speed were negatively correlated with each other during the aggregation and mound phases. These results suggest that cell migration does not require high levels of ATP or that the intracellular ATP levels are sufficiently high to support cell migration. The intracellular ATP levels of *D. discoideum* cells have been previously reported to be approximately 1–1.5 mM [63]. As the level of ATP is much higher than the critical level of submicromoles (~ 0.17 μM) required for the growth and shrinkage of a single actin filament [64], ATP levels may not be directly related to cell migration speed during the developmental process.

On the other hand, the analysis of the direction of cell migration in the mound indicated that ATP‐rich cells exhibit larger velocities to the mound center and smaller velocities in the tangential direction than ATP‐poor cells (Fig. 3). Our findings are consistent with the previous finding that prestalk cells migrate to the central tip region by centripetal movement [24]. Therefore, these results suggest that ATP‐rich cells constitute the prestalk cells via centripetal movements. Moreover, our simulation results suggest that a stronger central force of ATP‐rich cells could induce the aggregation of ATP‐rich cells to the mound center on their own, but the weaker contact‐following force could further promote efficient aggregation. The relative magnitude of each force can be speculated to be an important factor in determining cell movement during the mound phase. The collective migration of cells at the mound phase is an important process for cells to differentiate into either prespore or prestalk cells in *D. discoideum* [47, 65, 66, 67], playing a critical role in the formation of a distinct prestalk region in the central tip region of the mound [68]. In the mound phase, prespore cells migrate tangentially within the mound, whereas prestalk cells migrate toward the central tip region via centripetal movement [24]. These complex cell migrations during the mound phase are driven by cell–cell contacts [24, 47] and cell–cell signal transduction [47, 69, 70]. TgrB1/TgrC1 (E‐set Ig‐like domain‐containing heterophilic adhesion molecules) [24] and talin B [47] are adhesion molecules required for collective cell migration during the mound phase. In addition, when cells attach to each other, the high rates of polymerization of actin filaments are specifically observed at the leading edge of the follower cells [24]. This degree of polymerization was rarely observed in leader cells, which are likely to have a weaker contact force, suggesting that leader cells drive centripetal movement against collective cell migration of the surrounding cells. Moreover, migration of prestalk cells to the tip region is influenced by several Ras GTPases, including RasGAP1 [71], RasD [72], and RapC [73]. In particular, RapC null mutant cells tend to differentiate into stalk cells and have a stronger cell‐substratum contact, resulting in slower motility [73, 74]. The slower motility of ATP‐rich cells during the aggregation and mound phases as shown in Fig. 2B suggests that they are in close contact with the substratum. It has also been reported that cells displaying strong contact with the substratum tend to differentiate into stalk cells [75]. Thus, Ras GTPases may partially regulate the phenotype of ATP‐rich cells. The expression levels of adhesion molecules, actin polymerization levels, and the expression levels of Ras GTPases in ATP‐rich cells may be important for understanding the behavior of ATP‐rich cells during the mound phase; however, this remains unclear and will need to be elucidated further in future studies.

During morphogenesis, a small number of cells move in different directions from other cells, and such prominent movement of particular cells plays a critical role in morphogenesis, such as gonad formation [4]. Although it is not yet known whether these eccentrically migrating cells have higher ATP levels than other cells, our results may reflect this phenomenon. The dynamic reorganization of cytoskeletal filaments, such as myosin II‐dependent actin filament formation, which requires ATP hydrolysis, must occur continuously during morphogenesis [61, 76]. In addition, the expression of some genes related to morphogenesis and development is regulated by ATP‐dependent chromatin remodeling [77, 78]. Furthermore, in cancer invasion, which shows similar patterns of cell rearrangement with sprouting angiogenesis in the morphogenesis of various organs, the energetic regulation of the dynamic rearrangement of leader and follower cells has been reported, consuming large amounts of energy and inducing a transient decrease in the ATP/ADP ratio in leader cells, which leads to the subsequent replacement between leader and follower cells [79]. Therefore, understanding the behavior of migrating cells in the context of ATP levels is important for understanding the morphogenesis that accompanies cell migration.

## Conflict of interest

The authors declare no conflict of interest.

## Author contributions

HH, TN, YHiraoka, and TH conceived and designed the experiments. HH, JW, TN, YHirano, and SY performed experiments. HH, JW, TN, YHirano, YHiraoka, and TH analyzed the data. All the authors contributed reagents/materials/analysis tools. HH, TN, YHiraoka and TH wrote the manuscript with input from all the authors.

## Supporting information


**Fig. S1.** Typical development of *D. discoideum* from mound to slug.
**Fig. S2.** Evaluation of DicMaLionR as an ATP sensor probe.
**Fig. S3.** Cell distribution in the mound phase.
**Fig. S4.** Evaluation of DicMaLionR/flamindo2 cells.
**Fig. S5.** Changes in ATP and cAMP levels during development.

**Movie S1.** Related to Fig. S1. Typical development of *D. discoideum* from mound to slug.

**Movie S2.** Related to Fig. S4B. Typical time‐lapse images of living cells expressing flamindo2 during the aggregation phase.

**Movie S3.** Related to Fig. 4A. Simulation analysis of collective rotational motion. Initially, a total of 1000 cells, including 100 ATP‐rich cells (magenta) and 900 ATP‐poor cells (green), were distributed randomly in the circular region. The relative magnitude of central force ($w^{\text{cent}}$) and contact‐following force ($w^{\text{cont}}$) of ATP‐rich cells against ATP‐poor cells is 1.5 and 1.0, respectively. The number on the upper‐left side of each image indicates the time as “hours:minutes.”

**Movie S4.** Related to the upper left panel of Fig. 4C. The relative magnitudes of central ($w^{\text{cent}}$) and contract‐following forces ($w^{\text{cont}}$) on the locations of ATP‐rich cells (magenta) and their trajectories (yellow). ATP‐poor cells are shown in green. $w^{\text{cent}}$ = 1.0 and $w^{\text{cont}}$ = 1.0.

**Movie S5.** Related to the upper middle panel of Fig. 4C. $w^{\text{cent}}$ = 1.5 and $w^{\text{cont}}$ = 1.0.

**Movie S6.** Related to the upper right panel of Fig. 4C. $w^{\text{cent}}$ = 3 and $w^{\text{cont}}$ = 1.0.

**Movie S7.** Related to the lower left panel of Fig. 4C. $w^{\text{cent}}\;$= 1.0 and $w^{\text{cont}}$ = 0.5.

**Movie S8.** Related to the lower middle panel of Fig. 4C. $w^{\text{cent}}$ = 1.5 and $w^{\text{cont}}$ = 0.5.

**Movie S9.** Related to the lower right panel of Fig. 4C. $w^{\text{cent}}$ = 3.0 and $w^{\text{cont}}$ = 0.5.

## Data Availability

The authors declare that the data supporting the findings of this study are available within the paper and Supporting Information. Further data are available from the corresponding authors upon request.

## References

[1] Tahinci E , Lee E . The interface between cell and developmental biology. Curr Opin Genet Dev. 2004;14:361–6. 15261651 10.1016/j.gde.2004.06.013

[2] Garcia‐Garcia MJ , Anderson KV . Essential role of glycosaminoglycans in Fgf signaling during mouse gastrulation. Cell. 2003;114:727–37. 14505572 10.1016/s0092-8674(03)00715-3

[3] Dormann D , Weijer CJ . Chemotactic cell movement during *Dictyostelium* development and gastrulation. Curr Opin Genet Dev. 2006;16:367–73. 16782325 10.1016/j.gde.2006.06.003

[4] Richardson BE , Lehmann R . Mechanisms guiding primordial germ cell migration: strategies from different organisms. Nat Rev Mol Cell Biol. 2010;11:37–49. 20027186 10.1038/nrm2815 PMC4521894

[5] Marín O , Yaron A , Bagri A , Tessier‐Lavigne M , Rubenstein JLR . Sorting of striatal and cortical interneurons regulated by semaphorin‐neuropilin interactions. Science. 2001;293:872–5. 11486090 10.1126/science.1061891

[6] Devreotes P . *Dictyostelium discoideum*: a model system for cell‐cell interactions in development. Science. 1989;245:1054–8. 2672337 10.1126/science.2672337

[7] Firtel RA , Meili R . *Dictyostelium*: a model for regulated cell movement during morphogenesis. Curr Opin Genet Dev. 2000;10:421–7. 10889066 10.1016/s0959-437x(00)00107-6

[8] Loomis WF . The spatial pattern of cell‐type differentiation in *Dictyostelium* . Dev Biol. 1982;93:279–84. 6292026 10.1016/0012-1606(82)90117-8

[9] Kay RR . cAMP and spore differentiation in *Dictyostelium discoideum* . Proc Natl Acad Sci USA. 1982;79:3228–31. 6285342 10.1073/pnas.79.10.3228 PMC346388

[10] Williams JG . Transcriptional regulation of *Dictyostelium* pattern formation. EMBO Rep. 2006;7:694–8. 16819464 10.1038/sj.embor.7400714 PMC1500839

[11] Fukuzawa M . Control of prestalk‐cell differentiation by transcription factors. Dev Growth Differ. 2011;53:538–47. 21585358 10.1111/j.1440-169X.2011.01269.x

[12] Dormann D , Vasiev B , Weijer CJ . The control of chemotactic cell movement during *Dictyostelium* morphogenesis. Philos Trans R Soc Lond B Biol Sci. 2000;355:983–91. 11128992 10.1098/rstb.2000.0634 PMC1692793

[13] Chisholm RL , Firtel RA . Insights into morphogenesis from a simple developmental system. Nat Rev Mol Cell Biol. 2004;5:531–41. 15232571 10.1038/nrm1427

[14] Weijer CJ . *Dictyostelium* morphogenesis. Curr Opin Genet Dev. 2004;14:392–8. 15261655 10.1016/j.gde.2004.06.006

[15] Loomis WF . Genetic control of morphogenesis in *Dictyostelium* . Dev Biol. 2015;402:146–61. 25872182 10.1016/j.ydbio.2015.03.016 PMC4464777

[16] Konijn TM , van de Meene JG , Chang YY , Barkley DS , Bonner JT . Identification of adenosine‐3′,5′‐monophosphate as the bacterial attractant for myxamoebae of *Dictyostelium discoideum* . J Bacteriol. 1969;99:510–2. 4309098 10.1128/jb.99.2.510-512.1969 PMC250047

[17] Konijn TM , Chang Y‐Y , Bonner JT . Synthesis of cyclic AMP in *Dictyostelium discoideum* and *Polysphondylium pallidum* . Nature. 1969;224:1211–2. 4311588 10.1038/2241211a0

[18] Kriebel PW , Parent CA . Adenylyl cyclase expression and regulation during the differentiation of *Dictyostelium discoideum* . IUBMB Life. 2004;56:541–6. 15590560 10.1080/15216540400013887

[19] McMains VA , Liao X‐H , Kimmel AR . Oscillatory signaling and network responses during the development of *Dictyostelium discoideum* . Ageing Res Rev. 2008;7:234–48. 18657484 10.1016/j.arr.2008.04.003 PMC5155118

[20] Dormann D , Weijer CJ . Propagating chemoattractant waves coordinate periodic cell movement in *Dictyostelium* slugs. Development. 2001;128:4535–43. 11714678 10.1242/dev.128.22.4535

[21] Williams JG . *Dictyostelium* finds new roles to model. Genetics. 2010;185:717–26. 20660652 10.1534/genetics.110.119297 PMC2907197

[22] Kimmel AR , Firtel RA . Breaking symmetries: regulation of *Dictyostelium* development through chemoattractant and morphogen signal‐response. Curr Opin Genet Dev. 2004;14:540–9. 15380246 10.1016/j.gde.2004.08.001

[23] Williams HP , Harwood AJ . Cell polarity and *Dictyostelium* development. Curr Opin Microbiol. 2003;6:621–7. 14662359 10.1016/j.mib.2003.10.008

[24] Fujimori T , Nakajima A , Shimada N , Sawai S . Tissue self‐organization based on collective cell migration by contact activation of locomotion and chemotaxis. Proc Natl Acad Sci USA. 2019;116:4291–6. 30782791 10.1073/pnas.1815063116 PMC6410881

[25] Maeda Y , Maeda M . The calcium content of the cellular slime mold, *Dictyostelium discoideum*, during development and differentiation. Exp Cell Res. 1973;82:125–30. 4584618 10.1016/0014-4827(73)90253-x

[26] Saran S , Azhar M , Manogaran PS , Pande G , Nanjundiah V . The level of sequestered calcium in vegetative amoebae of *Dictyostelium discoideum* can predict post‐aggregative cell fate. Differentiation. 1994;57:163–9. 7988792 10.1046/j.1432-0436.1994.5730163.x

[27] Cubitt AB , Firtel RA , Fischer G , Jaffe LF , Miller AL . Patterns of free calcium in multicellular stages of *Dictyostelium* expressing jellyfish apoaequorin. Development. 1995;121:2291–301. 7671796 10.1242/dev.121.8.2291

[28] Baskar R , Chhabra P , Mascarenhas P , Nanjundiah V . A cell type‐specific effect of calcium on pattern formation and differentiation in *Dictyostelium discoideum* . Int J Dev Biol. 2000;44:491–8. 11032184

[29] Katz ER , Bourguignon LYW . The cell cycle and its relationship to aggregation in the cellular slime mold, *Dictyostelium discoideum* . Dev Biol. 1974;36:82–7. 4856682 10.1016/0012-1606(74)90192-4

[30] Zada‐Hames IM , Ashworth JM . The cell cycle and its relationship to development in *Dictyostelium discoideum* . Dev Biol. 1978;63:307–20. 147790 10.1016/0012-1606(78)90136-7

[31] Maeda Y , Ohmori T , Abe T , Abe F , Amagai A . Transition of starving *Dictyostelium* cells to differentiation phase at a particular position of the cell cycle. Differentiation. 1989;41:169–75. 2612767 10.1111/j.1432-0436.1989.tb00744.x

[32] Maeda Y . Regulation of growth and differentiation in *Dictyostelium* . Int Rev Cytol. 2005;244:287–332. 16157183 10.1016/S0074-7696(05)44007-3

[33] Maeda Y . Cell‐cycle checkpoint for transition from cell division to differentiation. Dev Growth Differ. 2011;53:463–81. 21585353 10.1111/j.1440-169X.2011.01264.x

[34] Leach CK , Ashworth JM , Garrod DR . Cell sorting out during the differentiation of mixtures of metabolically distinct populations of *Dictyostelium discoideum* . J Embryol Exp Morphol. 1973;29:647–61. 4736935

[35] Tasaka M , Takeuchi I . Role of cell sorting in pattern formation in *Dictyostelium discoideum* . Differentiation. 1981;18:191–6. 7327312 10.1111/j.1432-0436.1981.tb01122.x

[36] Matsuyama SI , Maeda Y . Involvement of cyanide‐resistant respiration in cell‐type proportioning during *Dictyostelium development* . Dev Biol. 1995;172:182–91. 7589798 10.1006/dbio.1995.0014

[37] Inazu Y , Chae SC , Maeda Y . Transient expression of a mitochondrial gene cluster including *rps4* is essential for the phase‐shift of *Dictyostelium* cells from growth to differentiation. Dev Genet. 1999;25:339–52. 10570466 10.1002/(SICI)1520-6408(1999)25:4<339::AID-DVG8>3.0.CO;2-3

[38] Chida J , Yamaguchi H , Amagai A , Maeda Y . The necessity of mitochondrial genome DNA for normal development of *Dictyostelium* cells. J Cell Sci. 2004;117:3141–52. 15226392 10.1242/jcs.01140

[39] Kimura K , Kuwayama H , Amagai A , Maeda Y . Developmental significance of cyanide‐resistant respiration under stressed conditions: experiments in *Dictyostelium* cells. Dev Growth Differ. 2010;52:645–56. 20887565 10.1111/j.1440-169X.2010.01200.x

[40] Hiraoka H , Nakano T , Kuwana S , Fukuzawa M , Hirano Y , Ueda M , et al. Intracellular ATP levels influence cell fates in *Dictyostelium discoideum* differentiation. Genes Cells. 2020;25:312–26. 32125743 10.1111/gtc.12763 PMC7318147

[41] Pitt GS , Milona N , Borleis J , Lin KC , Reed RR , Devreotes PN . Structurally distinct and stage‐specific adenylyl cyclase genes play different roles in *Dictyostelium* development. Cell. 1992;69:305–15. 1348970 10.1016/0092-8674(92)90411-5

[42] Rossomando EF , Sussman M . A 5′‐adenosine monophosphate‐ dependent adenylate cyclase and an adenosine 3′:5′‐cyelic monophosphate‐dependent adenosine triphosphate pyrophosphohydrolase in *Dictyostelium discoideum* . Proc Natl Acad Sci USA. 1973;70:1254–7. 16592080 10.1073/pnas.70.4.1254 PMC433470

[43] Rossomando EF , Ann‐Hesla M . Time‐dependent changes in *Dictyostelium discoideum* adenylate cyclase activity upon incubation with ATP. J Biol Chem. 1976;251:6568–73. 988025

[44] Arai S , Kriszt R , Harada K , Looi L‐S , Matsuda S , Wongso D , et al. RGB‐color intensiometric indicators visualize spatiotemporal dynamics of ATP in single cells. Angew Chem Int Ed Engl. 2018;57:10873–8. 29952110 10.1002/anie.201804304 PMC6456769

[45] Veltman DM , Akar G , Bosgraaf L , van Haastert P . A new set of small, extrachromosomal expression vectors for *Dictyostelium discoideum* . Plasmid. 2009;61:110–8. 19063918 10.1016/j.plasmid.2008.11.003

[46] Odaka H , Arai S , Inoue T , Kitaguchi T . Genetically‐encoded yellow fluorescent cAMP indicator with an expanded dynamic range for dual‐color imaging. PLoS ONE. 2014;9:e100252. 24959857 10.1371/journal.pone.0100252 PMC4069001

[47] Yamazaki S , Hashimura HV , Morimoto Y , Miyanaga Y , Matsuoka S , Kamimura Y , et al. Talin B regulates collective cell migration via PI3K signaling in *Dictyostelium discoideum* mounds. Biochem Biophys Res Commun. 2020;525:372–7. 32098673 10.1016/j.bbrc.2020.02.060

[48] Hashimura H , Morimoto YV , Yasui M , Ueda M . Collective cell migration of *Dictyostelium* without cAMP oscillations at multicellular stages. Commun Biol. 2019;2:1–15. 30701199 10.1038/s42003-018-0273-6 PMC6345914

[49] Meijering E , Dzyubachyk O , Smal I . Methods for cell and particle tracking. Methods Enzymol. 2012;504:183–200. 22264535 10.1016/B978-0-12-391857-4.00009-4

[50] Perfahl H , Hughes BD , Alarcón T , Maini PK , Lloyd MC , Reuss M , et al. 3D hybrid modelling of vascular network formation. J Theor Biol. 2017;414:254–68. 27890575 10.1016/j.jtbi.2016.11.013

[51] Umeda T , Inouye K . Possible role of contact following in the generation of coherent motion of *Dictyostelium* cells. J Theor Biol. 2002;219:301–8. 12419659 10.1006/jtbi.2002.3124

[52] Bretschneider T , Vasiev B , Weijer CJ . A model for cell movement during *Dictyostelium* mound formation. J Theor Biol. 1997;189:41–51. 9398502 10.1006/jtbi.1997.0490

[53] Driscoll MK , McCann C , Kopace R , Homan T , Fourkas JT , Parent C , et al. Cell shape dynamics: from waves to migration. PLoS Comput Biol. 2012;8:e1002392. 22438794 10.1371/journal.pcbi.1002392 PMC3305346

[54] Varnum B , Soll DR . Effects of cAMP on single cell motility in *Dictyostelium* . J Cell Biol. 1984;99:1151–5. 6088555 10.1083/jcb.99.3.1151 PMC2113391

[55] Yumura S . Reorganization of Actin and myosin II in *Dictyostelium* amoeba during stimulation by cAMP. Cell Struct Funct. 1993;18:379–88. 8033219 10.1247/csf.18.379

[56] Garcia R , Nguyen L , Brazill D . *Dictyostelium discoideum* SecG interprets cAMP mediated chemotactic signals to influence Actin organization. Cytoskeleton. 2013;70:269–80. 23564751 10.1002/cm.21107 PMC3693759

[57] Harwood AJ , Hopper NA , Simon MN , Bouzid S , Veron M , Williams JG . Multiple roles for cAMP‐dependent protein kinase during *Dictyostelium* development. Dev Biol. 1992;149:90–9. 1728597 10.1016/0012-1606(92)90266-j

[58] Firtel RA , Chapman AL . A role for cAMP‐dependent protein kinaseA in early *Dictyostelium* development. Genes Dev. 1990;4:18–28. 1968413 10.1101/gad.4.1.18

[59] Gancedo JM . Biological roles of cAMP: variations on a theme in the different kingdoms of life. Biol Rev. 2013;88:645–68. 23356492 10.1111/brv.12020

[60] Loomis WF . Cell signaling during development of *Dictyostelium* . Dev Biol. 2014;391:1–16. 24726820 10.1016/j.ydbio.2014.04.001 PMC4075484

[61] Lauffenburger DA , Horwitz AF . Cell migration: a physically integrated molecular process. Cell. 1996;84:359–69. 8608589 10.1016/s0092-8674(00)81280-5

[62] Zanotelli MR , Goldblatt ZE , Miller JP , Bordeleau F , Li J , VanderBurgh JA , et al. Regulation of ATP utilization during metastatic cell migration by collagen architecture. Mol Biol Cell. 2018;29:1–9. 29118073 10.1091/mbc.E17-01-0041 PMC5746062

[63] Roos W , Scheidegger C , Gerisch G . Adenylate cyclase activity oscillations as signals for cell aggregation in *Dictyostelium discoideum* . Nature. 1977;266:259–61. 191758 10.1038/266259a0

[64] Ranjith P , Mallick K , François Joanny J , Lacoste D . Role of ATP‐hydrolysis in the dynamics of a single Actin filament. Biophys J. 2010;98:1418–27. 20409460 10.1016/j.bpj.2009.12.4306 PMC2856186

[65] Kay RR , Thompson CRL . Forming patterns in development without morphogen gradients: scattered differentiation and sorting out. Cold Spring Harb Perspect Biol. 2009;1:a001503. 20457561 10.1101/cshperspect.a001503 PMC2882119

[66] Nicol A , Jan Rappel W , Levine H , Loomis WF . Cell‐sorting in aggregates of *Dictyostelium discoideum* . J Cell Sci. 1999;112:3923–9. 10547353 10.1242/jcs.112.22.3923

[67] Clow PA , Ling L , Chen T , Chisholm RL , McNally JG . Three‐dimensional in vivo analysis of *Dictyostelium* mounds reveals directional sorting of prestalk cells and defines a role for the myosin II regulatory light chain in prestalk cell sorting and tip protrusion. Development. 2000;127:2715–28. 10821769 10.1242/dev.127.12.2715

[68] Gregor T , Fujimoto K , Masaki N , Sawai S . The onset of collective behavior in social amoebae. Science. 2010;328:1021–5. 20413456 10.1126/science.1183415 PMC3120019

[69] Parkinson K , Bolourani P , Traynor D , Aldren NL , Kay RR , Weeks G , et al. Regulation of Rap1 activity is required for differential adhesion, cell‐type patterning and morphogenesis in *Dictyostelium* . J Cell Sci. 2009;122:335–44. 19126673 10.1242/jcs.036822 PMC2724730

[70] Bretschneider T , Othmer HG , Weijer CJ . Progress and perspectives in signal transduction, Actin dynamics, and movement at the cell and tissue level: lessons from *Dictyostelium* . Interface Focus. 2016;6(5):20160047. 27708767 10.1098/rsfs.2016.0047 PMC4992746

[71] Lee S , Escalante R , Firtel RA . A Ras GAP is essential for cytokinesis and spatial patterning in *Dictyostelium* . Development. 1997;124:983–96. 9056774 10.1242/dev.124.5.983

[72] Reymond CD , Gomer RH , Nellen W , Theibert A , Devreotes P , Firtel RA . Phenotypic changes induced by a mutated ras gene during the development of *Dictyostelium* transformants. Nature. 1986;323:340–3. 3093890 10.1038/323340a0

[73] Park B , Kim H , Jeon TJ . Loss of RapC causes defects in cytokinesis, cell migration, and multicellular development of *Dictyostelium* . Biochem Biophys Res Commun. 2018;499:783–9. 29614268 10.1016/j.bbrc.2018.03.223

[74] Jeon J , Kim D , Jeon TJ . Opposite functions of RapA and RapC in cell adhesion and migration in *Dictyostelium* . Anim Cells Syst (Seoul). 2021;25:203–10. 34413965 10.1080/19768354.2021.1947372 PMC8370755

[75] Sandrine A , Mathieu F , Silvia DM . Evolving social behaviour through selection of single‐cell adhesion in *Dictyostelium discoideum* . BioRxiv. 2021;1–23.

[76] Vicker MG , Grutsch JF . Dual chemotaxis signalling regulates *Dictyostelium* development: intercellular cyclic AMP pulses and intracellular F‐Actin disassembly waves induce each other. Eur J Cell Biol. 2008;87:845–61. 18554748 10.1016/j.ejcb.2008.03.010

[77] Curtis CD , Davis RB , Ingram KG , Griffin CT . Chromatin‐remodeling complex specificity and embryonic vascular development. Cell Mol Life Sci. 2012;69:3921–31. 22618247 10.1007/s00018-012-1023-4 PMC3661716

[78] Ye Y , Li M , Gu L , Chen X , Shi J , Zhang X , et al. Chromatin remodeling during in vivo neural stem cells differentiating to neurons in early *Drosophila* embryos. Cell Death Differ. 2017;24:409–20. 27858939 10.1038/cdd.2016.135 PMC5344203

[79] Zhang J , Goliwas KF , Wang W , Taufalele PV , Bordeleau F , Reinhart‐King CA . Energetic regulation of coordinated leader–follower dynamics during collective invasion of breast cancer cells. Proc Natl Acad Sci USA. 2019;116:7867–72. 30923113 10.1073/pnas.1809964116 PMC6475372

