# Supplementary material for: ATP levels influence cell movement during the mound phase in Dictyostelium discoideum as revealed by ATP visualization and simulation
Source: FEBS Open Bio. 2022 Sep 23;12(11):2042–56. doi: 10.1002/2211-5463.13480 (PMC9623536; doi:10.1002/2211-5463.13480)
Supplement: Supplementary file 1 — Fig. S1. Typical development of D. discoideum from mound to slug. Fig. S2. Evaluation of DicMaLionR as an ATP sensor probe. Fig. S3. Cell distribution in the mound phase. Fig. S4. Evaluation of DicMaLionR/flamindo2 cells. Fig. S5. Changes in ATP and cAMP levels during development. [file FEB4-12-2042-s002.pdf]

Supporting Information

ATP levels influence cell movement during the mound phase in *Dictyostelium discoideum* as revealed by ATP visualization and simulation

*Haruka Hiraoka[1,2], Jiewen Wang[3], *Tadashi Nakano[3], Yasuhiro Hirano[1], Shinichi Yamazaki[1], Yasushi Hiraoka[1], *Tokuko Haraguchi[1]

[1]Graduate School of Frontier Biosciences, Osaka University, 1-3 Yamadaoka, Suita, Osaka 565-0871, Japan
[2]Graduate School of Science, Nagoya University, 1 Furo-cho, Chigusa-ku, Nagoya, Aichi 464-8602, Japan
[3]Graduate School of Informatics, Osaka Metropolitan University, 3-3-138 Sugimoto, Sumiyoshi-ku, Osaka, Osaka 558-8585, Japan

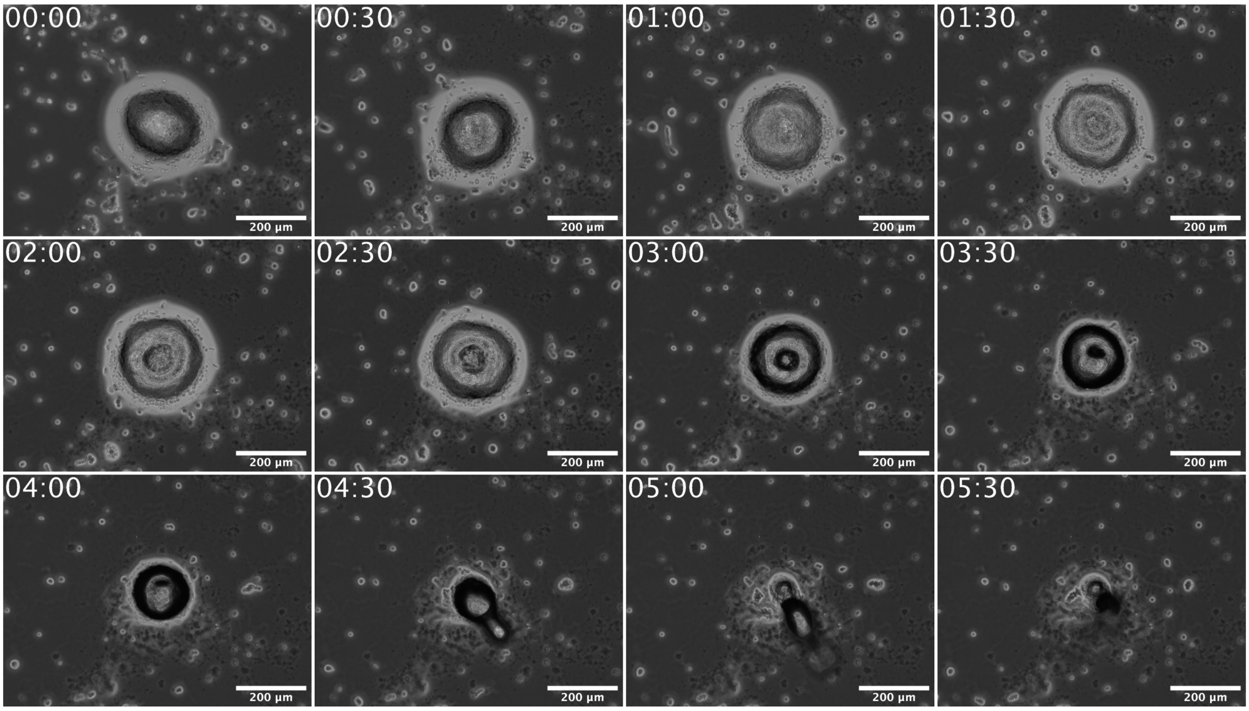

Supplementary Figure 1. Typical development of *D. discoideum* from mound to slug. Images were obtained using an Olympus IX83 microscope at 1-min intervals for 5.5 h, as described in Materials and Methods. Phase-contrast images from the mound to the slug phases are shown. The number on the upper-left side of each image indicates the time in "hours:minutes". Scale bar, 200 µm.

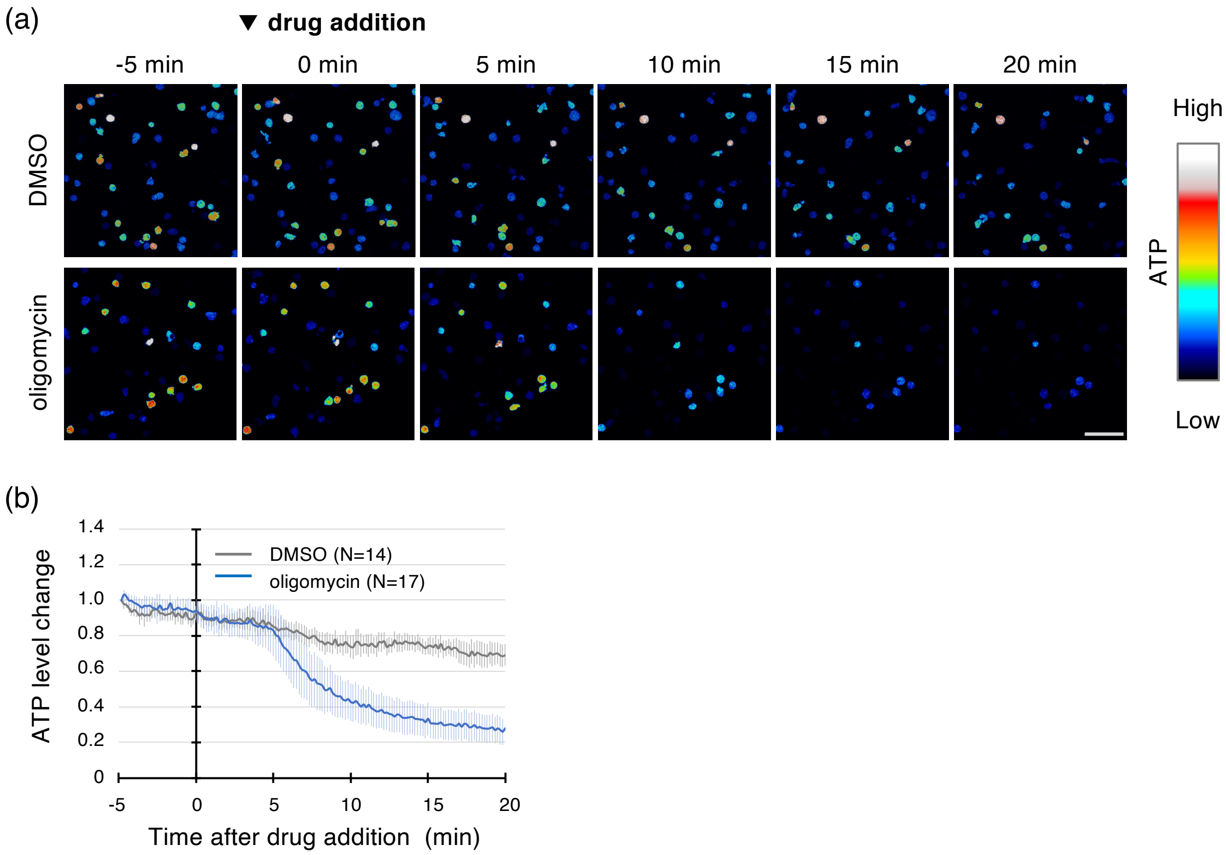

Supplementary Figure 2. Evaluation of DicMaLionR as an ATP sensor probe

(a) Fluorescence images of DicMaLionR. The vegetative cells expressing DicMaLionR were treated with oligomycin (lower panels), or dimethyl sulfoxide (DMSO) as a control solvent (upper panels), for the time periods indicated. The color scale on the right indicates ATP levels. Images were captured every 10 sec by Dragonfly200 using a 60× oil immersion objective lens (PlanApo 60×/1.40 NA, Nikon). Scale bar, 50 μm.

(b) The changes in DicMaLionR (ATP level) fluorescence intensities obtained in (a) were plotted against time following treatment with oligomycin or DMSO. Fluorescence intensities for individual cells were measured using ImageJ and plotted over time by setting the initial intensity (−5 min) to 1. The blue and gray lines represent the effects of oligomycin and DMSO treatment, respectively. The cell count (N) is as follows: N = 17 (oligomycin) and N = 14 (DMSO). The mean and standard deviation (SD) are shown.

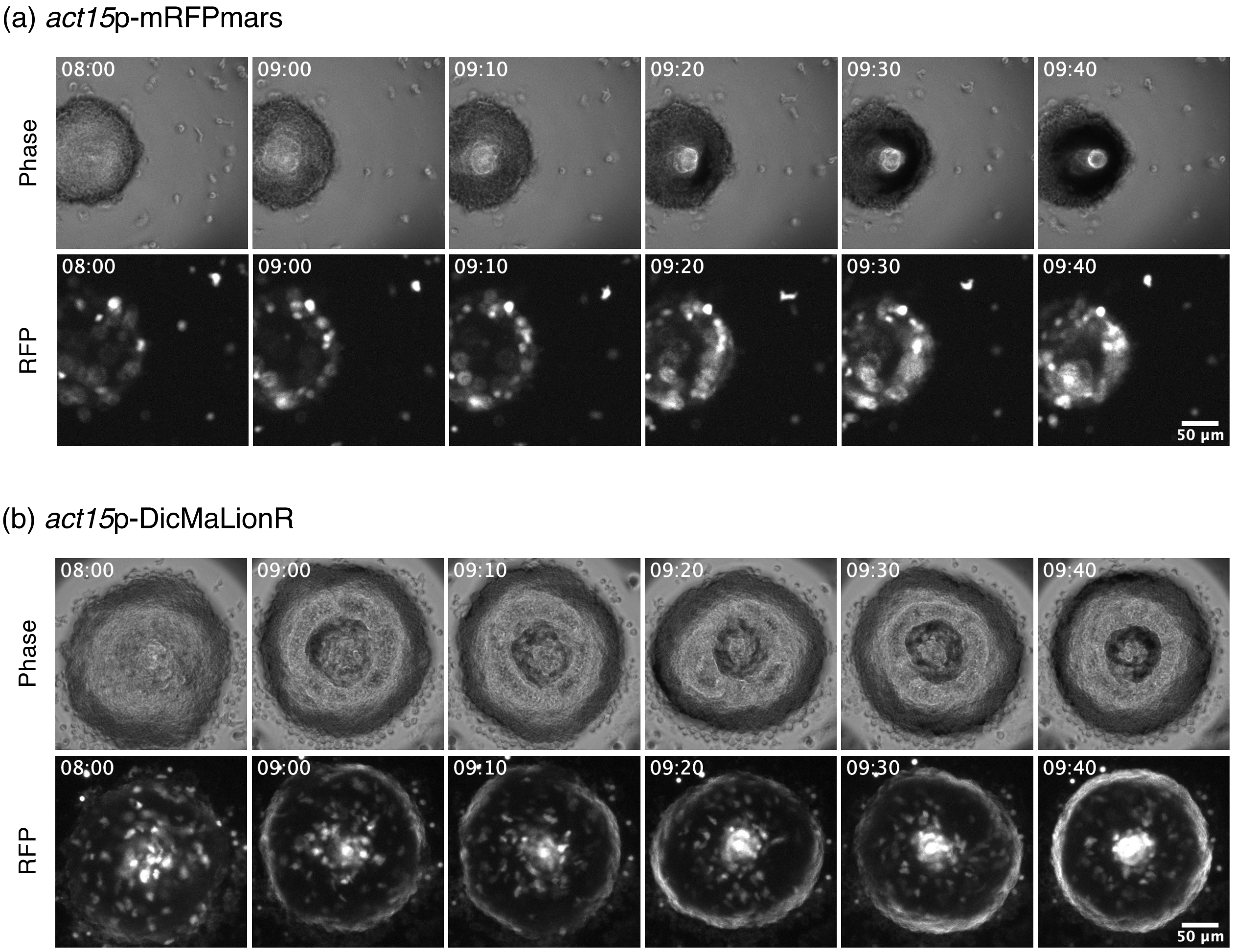

Supplementary Figure 3. Cell distribution in the mound phase.
Phase contrast and fluorescence images of live *D. discoideum* cells expressing either (a) mRFPmars or (b) DicMaLionR under the *act15* promoter. 5% of the cells expressing mRFPmars or DicMaLionR were mixed with unlabeled WT cells and allowed to develop. Time-lapse images were captured at 1 min intervals as described in the Materials and Methods section. The number on the upper-left side of each image indicates the time in "hours:minutes". Scale bar, 50 μm.

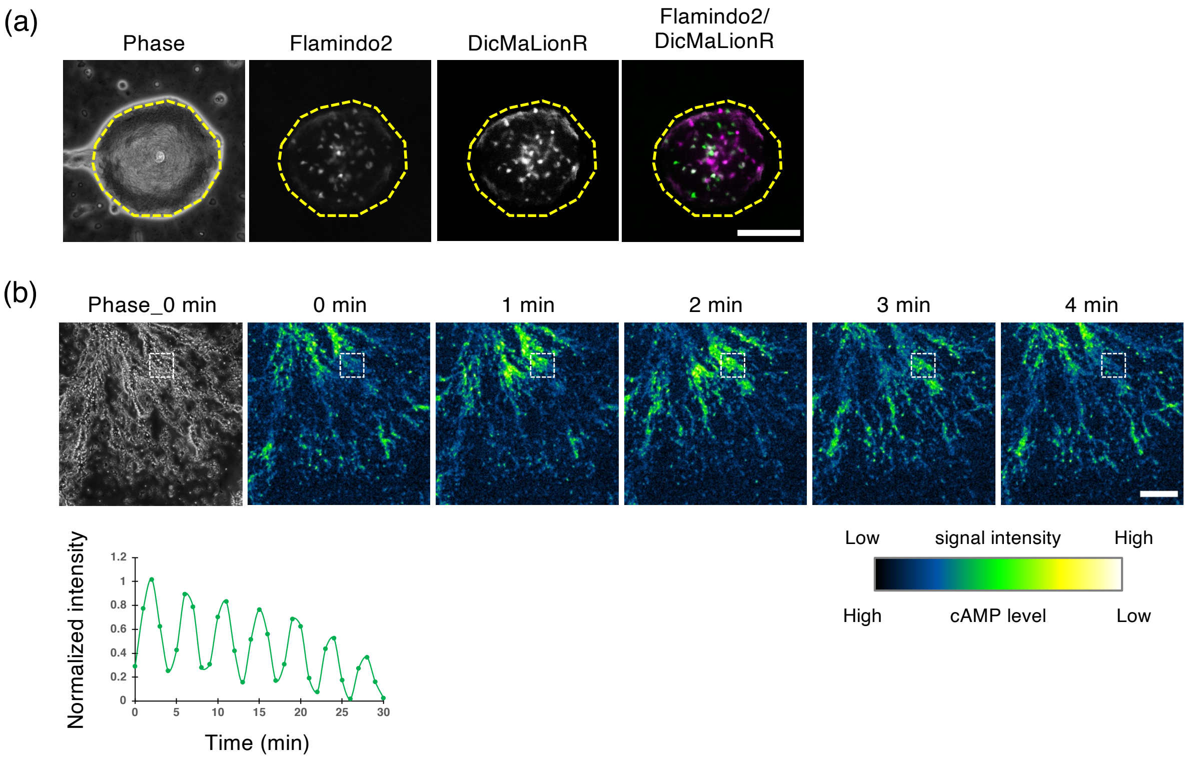

Supplementary Figure 4. Evaluation of DicMaLionR/flamindo2 cells

(a) Typical microscopic images of living cells expressing DicMaLionR/flamindo2 during the mound phase. Phase-contrast images (leftmost), fluorescence images of flamindo2 (second left), fluorescence images of DicMaLionR (third left), and merged images of flamindo2 and DicMaLionR (rightmost). Dashed white lines indicate the outline of the mound body. Scale bar, 100 µm. (b) Typical time-lapse images of living cells expressing flamindo2 during the aggregation phase. The leftmost image is a phase-contrast image at the start of observation. Time-lapse images were obtained every 1 min for 30 min. Images are shown for the selected time points. Fluorescence intensities in the boxed region were quantified using ImageJ software. The values were normalized with the maximum value as 1 and plotted as a function of time. Clear oscillatory of cAMP can be observed. Scale bar, 200 µm.

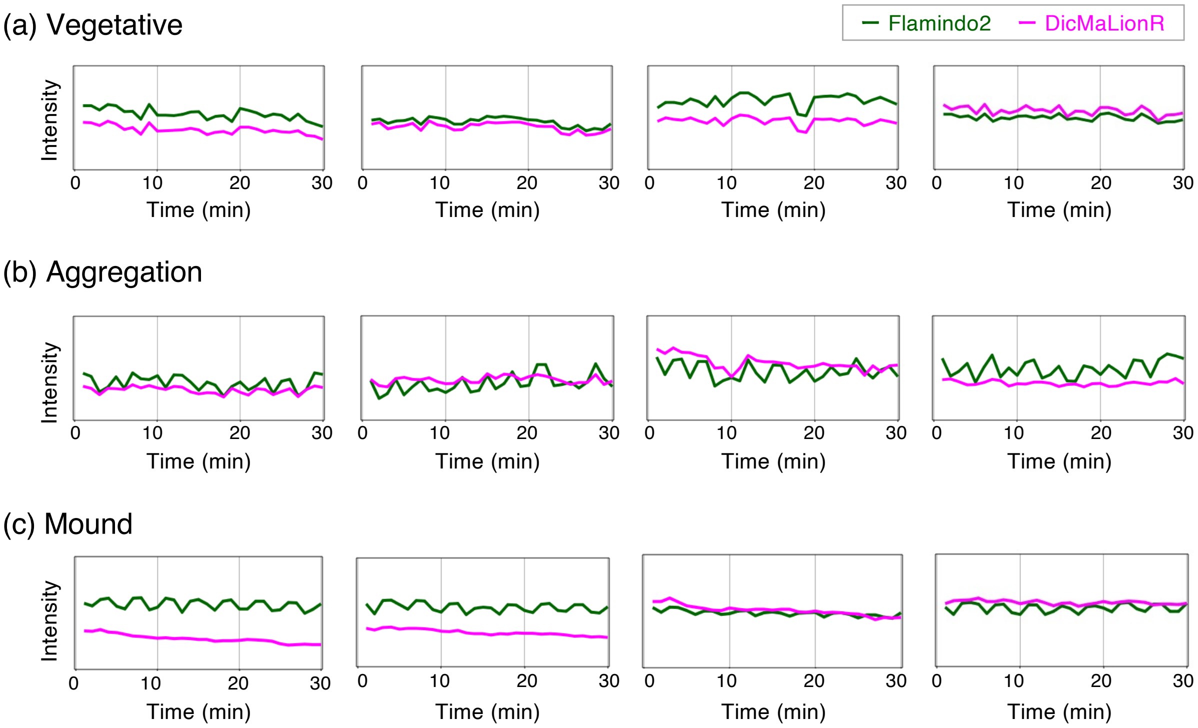

Supplementary Figure 5. Changes in ATP and cAMP levels during development. The fluorescence intensities of the single cells observed in Fig. 2 were quantified using ImageJ software: (a) vegetative, (b) aggregation, and (c) mound. Graphs indicate the time changes in the fluorescence intensities of flamindo2 (green) and DicMaLionR (magenta) in a single cell.
